# 203 Healthcare professional and commissioners’ perspectives on the factors facilitating and hindering the implementation of digital tools for self-management of long-term conditions within UK healthcare pathways

**DOI:** 10.1093/eurpub/ckae114.074

**Published:** 2024-09-26

**Authors:** James Gavin, Paul Clarkson, Paul Muckelt, Rachael Eckford, Euan Sadler, Suzanne McDonough, Mary Barker

**Affiliations:** School of Health Sciences, University of Southampton, Southampton, United Kingdom; School of Health Sciences, University of Southampton, Southampton, United Kingdom; NIHR Applied Research Collaboration Wessex, Southampton, United Kingdom; School of Health Sciences, University of Southampton, Southampton, United Kingdom; School of Health Sciences, University of Southampton, Southampton, United Kingdom; School of Health Sciences, University of Southampton, Southampton, United Kingdom; University of Medicine and Health Sciences, Royal College of Surgeons in Ireland, Dublin, Ireland; School of Health Sciences, University of Southampton, Southampton, United Kingdom; Faculty of Medicine, Southampton, United Kingdom

## Abstract

**Purpose:**

Physical activity is important in the self-management of long-term conditions (LTCs). However, implementing physical activity into clinical practice is challenging, due to complex barriers including access to programmes, time pressures, and transport costs, for people with comorbidities, managing multiple responsibilities. Various digital tools exist to overcome these barriers and support wide-scale implementation to help people stay physically active.

We explored the needs and preferences of healthcare professionals and commissioners, regarding the use of digital tools to support people with LTCs to self-manage using physical activity. This included barriers and facilitators to implementing digital tools to support people with LTCs in NHS settings.

**Methods:**

Semi-structured interviews were conducted (April 2021 to January 2022) in Wessex, southern England, UK. Purposive sampling was used to recruit general practitioners and healthcare professionals, whereas convenience sampling was used to recruit commissioners (n = 15). Transcripts were coded to develop conceptual themes allowing comparisons between and among perspectives, with the Normalisation Process Theory used to aid interpretation.

**Results:**

Results showed that most digital tools supporting physical activity for LTCs, are not well implemented clinically. Current digital tools were seen to lack condition-specificity, functionality, evidence-base, and voluntary sector involvement. Healthcare professionals and commissioners would not engage, unless digital tools were integrated into health service IT systems and professional networks, or adaptable according to the digital literacy of service users, and staff. For example, being technologically (easy to use) and culturally accessible (societal wide) to individuals. COVID-19 positively changed professional’s attitudes towards digital tools, in terms of being viable, feasible and critical options. Implementation was also influenced by endorsement and trustworthiness (i.e., security and evidence-based).

**Conclusion:**

Our findings highlight that consideration must be given to ensuring that digital tools are accessible to both healthcare professionals and patients, have functionality, and are adaptable to specific LTCs. To promote clinical engagement, digital tools must be evidence-based, endorsed by professional networks, and integrated into existing health systems. Digital literacy of patients and professionals is also crucial for cross-service implementation.

